# Experiences of end of life amongst family carers of people with advanced dementia: longitudinal cohort study with mixed methods

**DOI:** 10.1186/s12877-017-0523-3

**Published:** 2017-07-03

**Authors:** Kirsten J Moore, Sarah Davis, Anna Gola, Jane Harrington, Nuriye Kupeli, Victoria Vickerstaff, Michael King, Gerard Leavey, Irwin Nazareth, Louise Jones, Elizabeth L. Sampson

**Affiliations:** 1 Marie Curie Palliative Care Research Department, University College London, London, UK; 2 Division of Psychiatry, University College London, London, UK; 3 Bamford Centre for Mental Health & Wellbeing, University of Ulster, Magee Campus, Derry Londonderry, UK; 4 Department of Primary Care and Population Health, UCL Royal Free Site, London, UK

**Keywords:** Carer, End of life, Advanced dementia, Anxiety, Burden, Depression, Grief, Mixed methods

## Abstract

**Background:**

Many studies have examined the mental health of carers of people with dementia. Few have examined their experiences in the advanced stages of disease and into bereavement. We aimed to understand the experiences of carers during advanced dementia exploring the links between mental health and experiences of end of life care.

**Methods:**

Mixed methods longitudinal cohort study. Thirty-five family carers of people with advanced dementia (6 at home, 29 in care homes) were recruited and assessed monthly for up to nine months or until the person with dementia died, then at two and seven months into bereavement. Assessments included: Hospital Anxiety and Depression Scale, Short Form 12 health–related quality of life, 22-item Zarit Burden Interview, Brief Coping Orientation to Problems Experienced, Inventory of Complicated Grief and Satisfaction with Care at End of Life in Dementia. Subsequently, 12 carers (34%) were bereaved and 12 undertook a qualitative interview two months after death; these data were analysed thematically. We analysed quantitative and qualitative data independently and then merged findings at the point of interpretation.

**Results:**

At study entry psychological distress was high; 26% reached caseness for depression and 41% for anxiety and median complicated grief scores were 27 [IQR 22–37] indicating that on average 11 of the 16 grief symptoms occurred at least monthly. Physical health reflected population norms (mean = 50) and median burden scores were 17 [IQR 9–30]. Three qualitative themes were identified: the importance of relationships with care services, understanding of the progression of dementia, and emotional responses to advanced dementia. An overarching theme tying these together was the carer’s ability to control and influence end of life care.

**Conclusions:**

While carers report high levels of psychological distress during advanced dementia, the experience of end of life care in dementia may be amenable to change with the provision of sensitive and timely information about the natural progression of dementia. Regular health status updates and end of life discussions can help families understand dementia progression and prepare for end of life. The extent to which our findings reflect practice across the UK or internationally warrants further investigation.

## Background

Worldwide, 46 million people have dementia, a number expected to treble by 2050 [1]. Most die in long term care or in hospital [2, 3] often experiencing high symptom burden and medical interventions which may not be beneficial [4]. Informal (unpaid) and family carers contribute practical, emotional and financial support to their care. In the UK there are approximately 670,000 such carers and it is estimated that they save the state £11 billion per year [5]. The European Association for Palliative Care published recommendations for optimal palliative care for people with dementia and includes recommendations on meeting the needs of family carers covering burden, grief, and need for information and support throughout dementia [6].

Carers may find satisfaction and meaning in the caring role but also experience distress, depression, anxiety and guilt [7–9], often when they feel that they no longer have sufficient resources to cope [10]. This may be compounded by conflict between caring and other relationship and work roles, leading to a sense of being trapped within the caring role [11].

Grief has been defined as “a normal complex psychological and emotional reaction occurring in response to a significant loss.” [8] However, for some individuals the grief experience can be persistent and disabling; this has been termed complicated or prolonged grief [12]. Dementia can impact on communication and relationships, and families may begin grieving before their relative’s death. This grief has been described as equal in intensity and breadth to post-death bereavement [13]. Carer depression tends to remain constant before and after the death of the person with dementia [14].

Despite our growing theoretical appreciation of the carer experience, we know little about how carers experience end of life (EOL) care in dementia and how their practical and emotional needs are met. Carers who feel unprepared for the death may be more likely to experience depression, anxiety, and complicated grief symptoms [15]. A small number of qualitative studies have explored carers’ experiences after death. Inadequate information about dementia progression, EOL care and emotional support have been identified as influencing the carer experience [16–18].

### Aim

We aimed to understand the experiences of carers during advanced dementia, examining links with both mental health and experiences of EOL care. In this paper, we present quantitative and qualitative data from a prospective study of carers of people with advanced dementia who were followed before the death of their relative and into bereavement.

## Design and methods

### Study design

We conducted a longitudinal cohort study of people with advanced dementia and their carers in Greater London, UK as part of the Compassion programme [19]. We recruited carers of people with dementia living at home or in a care home with nursing home beds. People with dementia were aged 65 years or over, had a clinical diagnosis of dementia according to DSM-IV criteria, and a Functional Assessment Staging Scale grade 6e and above, indicating advanced stages of dementia (e.g. doubly incontinent, loss of ability to speak more than six words, ambulatory ability lost or can’t hold up head independently) [20]. Carers approached for participation included family members or friends in regular contact with the person with dementia, usually the next of kin or a key decision maker. Carers were able to speak sufficient English to complete questionnaires. Further details on the recruitment and consent process can be found elsewhere [19]. Data arising from the cohort of people with dementia are reported separately.

This is a mixed methods study with qualitative and quantitative data collected concurrently to enable triangulation of findings [21]. The use of validated measures enabled a standardised way of assessing carers’ mental health and satisfaction with end of life care. The use of qualitative interviews after death enabled an in-depth exploration of carers’ experiences of end of life care. Combining these two data sources, which were first analysed independently, provided a rich and more in-depth description of the carer experience at end of life.

### Quantitative data

At study entry we interviewed carers and documented their age, gender and relationship to the person with dementia. During a face to face interview, carers were given the option of self-completing the measures or completing them via structured interview. This process was repeated at study entry and monthly for up to nine months or until the person with dementia died. Monthly follow up ensured that the carer’s experience was captured during the month prior to the death of the person with dementia. If the person with dementia died during the study period, we assessed carers at two and seven months after the death [22]. Measures completed at each assessment included: the Hospital Anxiety and Depression Scale (HADS, cut-off ≥8 indicating clinically significant depression or anxiety) [23]; Short Form 12 health –related quality of life Survey (SF-12) [24]; Zarit Burden Interview (ZBI) 22- item version [25]; Brief Coping Orientation to Problems Experienced (Brief COPE) [26]; and Satisfaction with Care at EOL in Dementia (SWC-EOLD) Scale [27]. We also used the Inventory of Complicated grief (ICG) [28] modified to be suitable for carers before the death of their relative (ICG Short-Form Pre-Loss) as modified and obtained from the authors Mitchell et al. [22]. Measures collected at each assessment are shown in Table 1. Assessment 2 months into bereavement aimed to maximise participation by avoiding the immediate post-death period but occurring early enough for EOL care to be readily recalled. The seven month assessment was intended to examine longer term depression and general health of the carer in bereavement and because intense grief must occur for at least six months to be considered an indication of complicated grief [12].

**Table 1:** Outcome measures completed at each study assessment

Measure	Description	Study entry and monthly until death of relative	2 months after death	7 months after death
Hospital Anxiety and Depression Scale (HADS) [23]	Anxiety and depression subscales range 0–21 with cut-off of ≥8 indicating clinically significant depression or anxiety.	*√*	*√*	*√*
Short Form 12 health –related quality of life Survey (SF-12) [24]	Comprises “physical” and “mental” health related quality of life. Scores calculated as t-scores with a USA general population mean score set at 50 and scores higher than 50 indicating better health than the general population [24]. Questions refer to past four weeks.	*√*	*√*	*√*
Zarit Burden Interview (ZBI) 22- item version [25]	Score ranges from 0 to 88 with higher scores indicating higher carer burden. No cut-off score for clinically significant level of burden identified.	*√*		
Brief Coping Orientation to Problems Experienced (Brief COPE) [26]	28 items with 14 subscales (See Table 2) of which six are described as dysfunctional: self-distraction, denial, substance use, behavioural disengagement, venting and self-blame [46]. Each subscale has two items with a possible range of 2–8 with higher scores indicating higher use of this strategy.	*√*		
Inventory of Complicated Grief (ICG) Short-Form Pre-Loss version	The original ICG [28] was modified to be suitable for carers before the death of their relative as obtained from the authors Mitchell et al. [22]. Wording of items modified for pre-death assessments to refer to symptoms “since diagnosis/person’s illness” instead of “since death”. Sixteen items with a score range of 16–80 with higher scores indicating higher levels of grief. Carers asked to indicate how they had been feeling over the past month.	*√*		*√*
Satisfaction with Care at EOL in Dementia (SWC-EOLD) Scale [27]	Possible scores 10–40 with higher scores indicating greater satisfaction with EOL care. No cut-off score for high or low satisfaction.	*√*	*√*	

### Qualitative data

We invited bereaved carers to take part in in-depth qualitative interviews at a place of their choice 2-months after the death. Interviews were conducted in the carer’s home or at the care home where the person with dementia had resided. We used a topic guide derived from the literature and our wider programme of work in this field [19] to explore their overall experience of advanced dementia and EOL care including information about dementia progression and EOL received since diagnosis. A subset of questions focused on the care provided to the person with dementia such as “What support or services did [the person with dementia] receive towards the end?” Questions also explored physical, psychological, spiritual and social needs and “how well were they dealt with by hospital/nursing home staff?” Another set of questions explored communication about end of life care such as “Did any staff (GP, Consultant, nursing home staff etc) talk to you about what might have happened to [the person with dementia] in the future?” “Did anyone discuss with you what the course of [person with dementia’s] illness was likely to be?” and “Was there any information regarding [the person with dementia’s] illness/care you found difficult to understand?” A further set of questions explored carers’ personal reaction to their relative’s illness, end of life care and their bereavement, for example: “How did you find dealing with [the person with dementia’s] illness” and “Have you had any help or support with your bereavement?”

### Data analysis

In this mixed methods study we analysed the quantitative and qualitative data independently with a statistician analysing the quantitative data (VV) and qualitative researchers analysing the qualitative interviews (KM, NK, SD and JH). We gave equal weighting to both sources of data and the point of integration was during interpretation and final write-up of results [21]. This approach aimed to reduce the impact of confirmation bias in analysing the two data sources. In the results section we present the quantitative data as stand-alone findings but for the qualitative data we have reflected on quantitative findings that contradict or support the qualitative findings when evident.

### Quantitative data

We used descriptive analyses, means or medians for continuous variables and frequencies for categorical variables to examine the data. For this analysis, we have presented data at 4 time points: 1) study entry; 2) the final assessment that the carer undertook while their relative with dementia was alive; either at the end of the 9 month study period or, if the relative died before this, the final assessment in the month before their death; 3) 2 months after the death of the person with dementia 4) 7 months post death. The SF-12 score is calculated as a t-score using a general population mean score based on a USA sample. Therefore the score is comparable to a general population score set at 50 with scores higher than 50 indicating better health than the general population and lower scores indicating poorer health [24].

### Qualitative data

Interviews were audio-recorded and transcribed verbatim then checked by a second researcher against the audio-recording. One researcher (KM) read and re-read the checked transcripts to familiarise herself with the data. Transcripts were thematically and inductively analysed to determine core themes identified in relation to being a carer during the advanced stages of dementia. NVivo V11 © QRS International qualitative software was used for coding chunks of text into nodes. Coding began with the first transcript and involved labelling chunks of text at new nodes. As related nodes were identified they were grouped together under broader themes. After completing coding for the first interview KM checked all nodes and tried to refine them by merging duplicating nodes and moving nodes under themes. KM continued this process for each transcript continuing to refine and regroup the themes. After half the transcripts had been reviewed fewer new nodes were identified, suggesting saturation of data was reached [29]. After coding was complete KM reviewed the first 3 interviews that had been coded to ensure that more recently added themes had not been initially missed. Themes were reviewed and refined further aiming to group related concepts and reduce duplication.

The NVivo file and summary were reviewed by a second researcher to check that all core themes were represented in the text (NK). SD and JH who had undertaken carer interviews and had read transcripts examined themes to ensure they “rang true” with data they had collected and reviewed.

For brevity we have not reported themes relating to the experiences of earlier stages in the progression of dementia nor have we reported themes well documented in the literature including care as a family responsibility and impacting on other family and employment responsibilities [30–32]. The remaining themes are summarised here.

### Ethics

Carers gave signed informed consent for their participation. The study was approved by the National Research Ethics Service Committee East of England – Essex (12/EE/0003).

## Results

### Participants

**Fig. 1:**
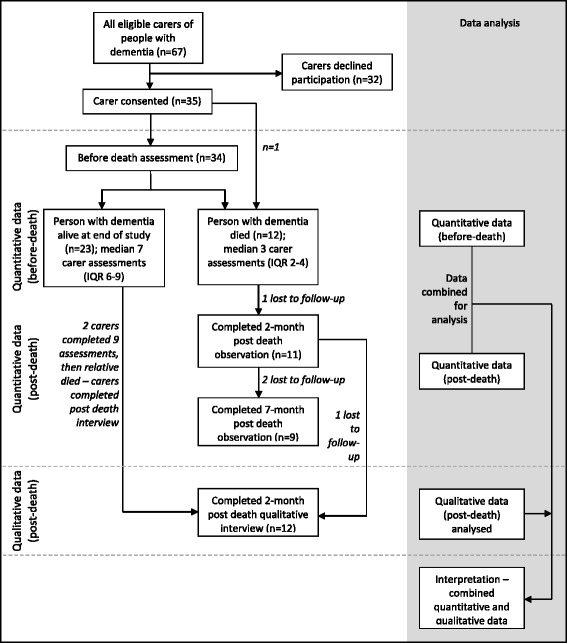
Flow chart of participating carers

Quantitative and qualitative data were collected concurrently between May 2012 and December 2014. Eighty-five people with advanced dementia were recruited to the cohort study of whom 67 had a family carer. Thirty-five carers (52% response rate) agreed to participate (Fig. 1), including six caring for someone at home and 29 caring for someone residing in a care home. Carers’ median age was 62 years (IQR: 54–69) and 24 (69%) were female; most were the daughter or son of the person with dementia (69%); seven were spouses (20%) and four were other relatives (11%). At study entry 58% of carers were married and 38% were in paid employment; one carer subsequently took early retirement. On average they provided assistance with eating, toileting and dressing ten days per month for 1.4 h on each day. One carer did not complete any pre-death assessments as their relative died between the time the carer consented and their first assessment was completed – they therefore only have the 2 and 7 month post death assessments. Twelve carers (34%) were bereaved during the observation period and completed measures at 2 months (*n* = 11) and 7 months (*n* = 9) after the death. Ten of these carers also completed a qualitative in-depth interview 2 months after the death. Another two carers completed qualitative interviews two months after their relative’s death, however, they have no post death quantitative data as their relative died shortly after the 9 month study period and therefore they are included in “non-bereaved” at final assessment in Table 2.

**Table 2:** Carer outcomes at study entry, last visit, and 2 and 7 months post death

	All carers (*n* = 35)		Non-bereaved carers (*n* = 23)	Bereaved carers (*n* = 12)		
	Study entry (*n* = 34)	Final assessment with person with dementia^a^ (*n* = 34)	Final assessment with person with dementia^a^ (*n* = 23)	Final assessment with person with dementia^a^ (*n* = 11)	2 months post death (*n* = 11)	7 months post death (*n* = 9)
ZBI, median (IQR)	17 (9–30)	11 (6–17)	11 (6–18)	11 (4–16)	-	-
Brief COPE mean (SD)						
Use of instrumental support	2.3 (0.8)	2.3 (0.7)	2.2 (0.5)	2.4 (0.9)	-	-
Substance use^b^	2.4 (0.7)	2.4 (0.8)	2.4 (0.8)	2.5 (0.8)	-	-
Denial^b^	2.5 (1.1)	2.3 (0.7)	2.3 (0.8)	2.2 (0.4)	-	-
Planning	2.9 (1.3)	2.8 (1.5)	3.2 (1.7)	2.0 (0.0)	-	-
Behavioural disengagement^b^	3.3 (1.5)	2.9 (1.2)	3.0 (1.2)	2.5 (1.0)	-	-
Religion	3.3 (1.7)	3.3 (1.8)	3.4 (1.9)	3.2 (1.5)	-	-
Venting^b^	3.5 (1.4)	3.3 (1.6)	3.3 (1.8)	3.2 (1.3)	-	-
Self-distraction^b^	3.7 (1.7)	3.2 (1.4)	3.2 (1.5)	3.3 (1.3)	-	-
Positive reframing	3.7 (1.9)	3.5 (1.8)	3.2 (1.5)	4.2 (2.3)	-	-
Self-blame^b^	3.7 (1.9)	4.0 (1.9)	4.0 (1.6)	3.8 (2.4)	-	-
Active coping	3.9 (2.2)	3.5 (1.6)	3.1 (1.2)	4.3 (2.0)	-	-
Acceptance	4.0 (2.4)	3.9 (2.3)	3.6 (2.1)	4.5 (2.7)	-	-
Use of emotional support	4.7 (1.9)	4.8 (2.0)	4.7 (2.1)	4.9 (1.9)	-	-
Humour	6.5 (1.8)	6.5 (2.1)	6.5 (2.0)	6.5 (2.3)	-	-
SWC-EOLD, median (IQR)	30 (26–34)	30 (29–33)	30 (29–33)	29 (28–35)	31 (25–37)	-
ICG, median (IQR)	27 (22–37)	24 (18–33)	22 (16–35)	27 (20–32)	-	23 (19–29)
HADS, *n =* n (%) cut off ≥8	*34*	*34*	*23*	*11*	*10*	*8*
anxiety	14 (41)	11 (32)	8 (35)	3 (27)	3 (30)	2 (25)
depression	9 (26)	7 (21)	5 (21)	2(18)	4 (40)	1 (13)
Both anxiety and depression	7 (20)	6 (17)	4 (17)	2 (18)	3 (30)	1 (13)
SF-12 *n=*	*32*	*33*	*22*	*11*	*11*	*9*
Physical component mean (SD)	50.32 (8.94)	50.73 (9.01)	50.15 (9.36)	51.88 (6.22)	48.18 (12.26)	46.40 (11.63)
Mental component mean (SD)	47.57 (11.08)	46.61 (11.87)	47.07 (11.98)	45.70 (11.36)	44.88 (15.29)	52.52 (9.74)

^a^The last assessment that the carer undertook while their relative with dementia was alive. For non-bereaved carers this would have been at the end of the 9 month study period, while for bereaved carers this would be ﻿the final assessment in the month before their relative died within the 9 month study period

^b^Dysfunctional coping strategies; Brief COPE = Brief Coping Orientation to Problems Experienced; SD = Standard Deviation; SF-12 = Short Form 12; ZBI = Zarit Burden Interview; IQR = Interquartile Range; HADS = Hospital Anxiety and Depression Scale; ICG = Inventory of Complicated Grief; SWC-EOLD = Satisfaction with Care at EOL in Dementia Scale

Eight daughters, two sons and two husbands participated in qualitative interviews. With the exception of two daughters who cared for a parent at home until their death (ID9, ID10), the remaining participants cared for someone who resided in a care home. The age range of these carers was 43–91, with half aged 50–64 years.

### Quantitative findings

We found high levels of emotional distress amongst carers both prior to and after the death of their relative. Rated on the HADS scale, between 18 and 26% of carers experienced clinically significant depression (scored ≥8) and 27–41% anxiety (scored ≥8) over the course of the study. Most carers who experienced depression also experienced anxiety. HADS scores at 2-months post-death indicated that 30% of carers reached caseness for depression and 40% for anxiety (Table 2). The SF-12 mental health subscale scores also indicated that carers’ mental health was consistently poorer over the study period than the USA sample population mean used for developing the SF-12 [24]. This remained 2-months post death but mental health was higher than population norms for those who took part in the 7-month post-death assessment.

On the ICG, median scores ranged between 22 and 27 with higher scores at study entry for all participants and at the final assessment before death for bereaved carers (Table 2). For the nine bereaved carers who completed the grief score at 7-months post death, scores were lower than their last assessment before death. At study entry 38% of carers scored at least 32 on the ICG. While there is no established clinical cut-off of this modified version of the ICF, a score of 32 indicates that on average carers reported all 16 items as occurring at least monthly rather than less than once a month indicating a high occurrence of grief symptoms. This proportion was 35% for non-bereaved carers at their final assessment. For bereaved carers 27% at the final assessment before death and 22% at the 7 month post death assessment scored 32 or above on the ICG.

Median ZBI scores were 17 at study entry; and decreased during follow up. Data on the Brief COPE showed less use of the coping strategies: instrumental support, substance use, denial and planning. The most commonly used strategy was humour, followed by seeking emotional support and acceptance. SF-12 scores indicated that carers’ physical functioning were comparable to population norms (mean = 50), although those who completed post-death assessments had scores below population norms. See Table 2 for scores at each time point.

Median SWC-EOLD scores remained stable (at 29–31) over time (Table 2). At least 30% of participants agreed with questions “I would probably have made different decisions if I had had more information” and “I feel that my relative needed better medical care at the end of his or her life” and 30% disagreed that they “felt fully involved in all decision making” (Table 3).

**Table 3:** Responses to the Satisfaction with care at the EOL in Dementia questionnaire, first assessment (*n* = 34)

	Strongly disagree (%)	Disagree (%)	Agree (%)	Strongly agree (%)
I felt fully involved in all decision making	2 (6)	8 (24)	16 (47)	8 (24)
I would probably have made different decisions if I had had more information	7 (21)	17 (50)	5 (15)	5 (15)
All measures were taken to keep my relative comfortable	0 (0)	3 (9)	17 (50)	14 (41)
The health care team was sensitive to my needs and feelings	2 (6)	4 (12)	20 (59)	8 (24)
I did not really understand my relative’s condition	15 (44)	16 (47)	3 (9)	0 (0)
I always knew which doctor or nurse was in charge of my relative’s care	2 (6)	7 (21)	19 (56)	6 (18)
I feel that my relative got all necessary nursing assistance	0 (0)	6 (18)	14 (41)	14 (41)
I felt that all medication issues were clearly explained to me	0 (0)	8 (24)	20 (59)	6 (18)
My relative received all treatments or interventions that he or she could have benefited from	0 (0)	8 (24)	19 (56)	7 (21)
I feel that my relative needed better medical care at the end of his or her life	4 (12)	19 (56)	8 (24)	3 (9)

### Qualitative findings

Three distinctive core themes were identified from our analysis of the interviews that appear specific to experiences of caring near the end of life in dementia: the importance of relationships with health and social care services, understanding of the progression of dementia, and emotional responses to advanced dementia and EOL care. We elaborate on these themes below. We then examine an overarching theme that appears to tie these three themes together – the carer’s perception of their ability to control and influence end of life care.

### The importance of relationships with health and social services

Carers often held strong views regarding the perceived quality of care. Five carers moved their relative from one service to another (3 from a care home and 2 from home-based care provision) when they were dissatisfied with the quality of care.

So I got back to her care manager and said “this isn’t on… give me lists of all other homes. I’m going to find her a better home and I’m going to complain formally about this particular care home because they should not be EMI [Elderly Mentally Impaired] registered”… I went around and found her another care home and in June 2010 she moved… and they were absolutely brilliant. (ID11, daughter)

Carers valued continuity, a safe environment, treating the person with dementia as a person and with dignity, and receiving regular feedback about their relative’s health condition and the progression of dementia.

[Care home staff] were very good that they sort of found out different ways to help her… and they always kept us informed of what they’d found better for mum. Because they obviously saw her all the time, so that was very good we felt. (ID2, daughter)

Carers were comforted when they felt that care staff genuinely cared for their relative and were trustworthy.

It wasn’t until she went into the final care home in June 2010 that I could relax about her care, because, as I said, they had the best provision for it, and I felt she was treated as an individual. (ID11, daughter)

Being able to monitor services was important and reflected poor levels of trust in service providers. One carer received input from the warden in the block of flats who would take note of when social services staff would visit for only a few minutes. Some carers monitored services through regular visits to the care home.

I knew if you’d stopped going up then like the standards would drop. When they knew I was coming up, I knew my Mum was always like kept spotless, if they knew I was there, everything would be near enough right. (ID5, son)

### Understanding of the progression of dementia

Carers’ capacity to understand the progression of dementia and be involved and informed during advanced dementia relied on information provision throughout the different stages of dementia. At diagnosis, carers were rarely informed about the likely progression of dementia.

Nobody said anything about the Alzheimer’s ever. Maybe they don’t know what... how it’s going to proceed. It was never really an issue any more. It was a label that had been given her and if she went into hospital it was mentioned. (ID10, daughter)

The unpredictable course of dementia made it very challenging for carers to prepare for EOL and some were unsure about the value of early information about advanced stages of disease given the potentially unnecessary anxiety this might create.

I don’t know whether it [information about symptoms of advanced dementia] would’ve helped or not actually. I mean it’s nothing I could have done anything about… … I think it might have helped yes. Might have made me more fearful of the future of course. (ID8, husband)

Findings also supported timely and sensitive information provided by a knowledgeable professional and that was reinforced in writing. Some felt that the lack of basic information left them struggling to adapt to changes and feeling ill-prepared for symptoms that they later discovered were common in advanced dementia:

Nobody really explained to me then when she was admitted into [Hospital] A&E. I said what is the cause of this foaming at the mouth? … I found out later it’s quite a common symptom of end stage dementia. (ID9, daughter)

If I’d have had a session with me and someone at the beginning, face to face, to say “right your mum has been diagnosed; this is what you need to expect”. But in a very gentle kind of... Like the leaflet you showed me that time. That would’ve been brilliant… being ignorant isn’t going to save you in the long term… need to know that this is a terminal illness, it’s not going to just “oh yeah they’re old they’ll go on till they’re a hundred but they’ll just have Alzheimer’s”. …maybe my sister wouldn’t have emigrated had she realised “look I’ve got mum in a home now…right I’m off.” (ID11, daughter)

EOL plans were rarely initiated during the early stages of dementia preventing the person with dementia being involved in decision making. Sometimes the person with dementia was never informed of their diagnosis. EOL planning often occurred after admission to a care home or after a critical health event usually involving hospitalisation in the advanced stages of dementia. Carers often appreciated these conversations as they could be involved in care and feel that they had contributed to a plan to promote comfort care at EOL. However, even when carers were well informed and prepared, a crisis could test these goals for comfort care as the carer was conflicted with wanting to keep their relative alive.

… the four paramedics stormed in, took over completely, and sent me and my sister out into the hallway… My sister couldn’t watch she was getting so upset and crying her eyes out because they literally pushed everything out of the way, threw her onto the floor, and she’s so frail... I don’t know whether they cracked her ribs and I was just standing there calmly watching them and saying to my sister “there’s still a chance.” But it never occurred to me to ask them to stop because we did agree on DNR but in the heat of that moment when you call 999 and when they ask you to do CPR you just do it. You know, it’s your mum; you try to revive her. (ID9, daughter)

Some carers were satisfied with EOL care if they felt adequately informed and involved, even when EOL care was not in accordance with advance care plans. One carer indicated that her father was not for resuscitation or hospitalisation but at the end of his life he was taken to hospital and experienced numerous investigations for suspected pneumothorax. She described the hospital staff as “brilliant” as they kept her informed. Other examples of potentially burdensome interventions were seen as necessary by carers, particularly when the intervention had been successful in the past and the person with dementia had recovered:

They said on one occasion she could be treated here [care home] with oral antibiotics or she could go to hospital and have intravenous antibiotics. And I quickly plumped for the hospital and they sorted her out in a day or two. She may not have recovered if she’d stayed here. That was maybe a year or two before she died. (ID7, husband).

There were many examples of comfortable EOL care and these occurred across hospital, home and care home settings. For most, but not all, being present at EOL was important and some described vigils from hours to weeks, being with the person before they died.

[*So who fed your mum?]* I used to go over and, the last few weeks I used to go over four times a day… Because her lips were cracked and she, she liked tasty bits… I’d sit and feed those to her. And she would eat them. (ID6, daughter)

For some, the difficulty in determining the exact time of death prevented being present at the time of death. Specialist palliative care was requested for some people in hospital; however, this was often described as occurring too late or after demands from the family. Once commenced, specialist palliative care was viewed as enabling a comfortable death and minimising distressing symptoms.

### Emotional responses to advanced dementia and EOL

While carers often described how well they coped with their relative’s dementia and dying, there were also many accounts that supported the quantitative findings of high levels of grief and distress. Carers described grief as a staged process pre and post death with losses associated with dementia before death:

It’s sort of two bereavements really isn’t it… first time when he moved out and went into a home... that was more traumatic to be honest… I was crying for about two weeks on and off when that happened and also I suppose it’s because you felt inadequate as well that you couldn’t cope with it. So this time, although you’ve lost them, before you’d lost the person he was. (ID4, daughter)

There was evidence of links between satisfaction with EOL care, emotional consequences and the carer’s capacity to influence the care being provided. Two carers who had not moved their relative from what they perceived as a poor quality care home, reported the lowest satisfaction on the SWC-EOLD and reported high ICG scores 7 months post death (scored 29 and 45). This was influenced by their guilt at not having done more to improve EOL care:

I’m not making excuses, you know, I could have been more proactive… I just feel I wish I’d done more for him really. I just think he deserved better and I hope that guilt feeling… that I’ll learn to live with that really. (ID12, daughter)

It’s left us burdened with the care system like this… I’d have turned our dining room into a bedsit for her regardless of what the doctors said. God yes, my husband cries about it. He gets so distressed. For months since she died I find it very hard to sleep. I feel I let her down… (ID6, daughter)

Participants discussed the failure of services to acknowledge their grief or to provide information about obtaining support. This was both prior to and after their relative’s death.

As soon as mum died... we had no meetings with social services or no communication even… We did find that strange that there wasn’t even a letter saying “sorry what’s happened”, you know, there was nothing… that would have been nice really to have a bit of continuing help for you or just to say “we’re here, this is where you could go for help, counselling or whatever or you might need it in the future”. (ID2, daughter)

Despite high levels of grief, many carers felt they did not need formal support or counselling and did not seek it. Instead they described the benefits of their social network including friends, family or faith community. Some carers could not face their grief or the fact that their relative had dementia. For one this led to episodes of binge drinking. Another explained:

I suppose we had been told a bit [about dementia] and we knew from what we see on the news and maybe you don’t want to accept it… for me personally maybe I pushed it to the back…didn’t want to believe it was happening. It was so awful I just didn’t want to believe that it was happening. (ID3, daughter)

### Carer’s perception of control

The three themes above are tied together by an overarching theme regarding the carer’s perception of their ability to control and influence EOL care. The findings above indicate that carers who felt well informed about how dementia progressed, were regularly updated on their relative’s health condition and felt involved appeared more satisfied with EOL care. Those who failed to influence care that they perceived as poor reported high levels of grief after death and experienced guilt and regret. Admission to a care home was often associated with a loss of control and a need for heightened vigilance. One carer whose mother remained at home until her death stated:

I have had experience with care homes I suppose with my mother-in-law, I suppose they weren’t great. But I’m sure there are good ones if you can afford them, but you don’t have the chance to see what they’re really like, you can’t spend time there. And also, the big issue was that you lost control. If you go into a home you don’t know whose looking after them, what’s happening at night when the main staff are away. (ID10, Daughter)

Another carer who described the importance of exerting control in the care home indicated:

I was pretty lucky because where I’ve always probably had people work for me for like 20 odd years, I was used to being in control. So I could go “well I want that done, that done, this done, that done.” And I sort of worked the same way with me mum. (ID5, son)

## Discussion

The Compassion research programme is one of the first studies to prospectively collect outcome data on carers up to the last month of life of their relative with advanced dementia and into bereavement. Combining quantitative and qualitative findings provided a rich understanding of their experiences over time.

### Carer burden and coping

Our sample, largely of carers with relatives in care homes, reported lower levels of burden compared to carers of people with dementia living in their own homes [33, 34]. The transition period to a care home is generally stressful, often following a crisis, and carers tend to receive limited information and emotional support [35]. It is likely that after a settling-in period, carer stress decreases if they feel the care home is providing an acceptable quality of care. This may explain the lower burden in this study where half the carers cared for someone resident in a care home for at least three years. Carers generally reported low use of all types of coping strategies. The relative importance of humour as a coping strategy compared with other strategies was evident and has been highlighted in other research [36]. Although the sample is too small to make statistical comparisons, it appears that in the last month of life carers were more likely to use positive reframing, acceptance and active coping while non-bereaved carers were more likely to be involved in planning at their last assessment.

### Carer mental health

While burden was low, our sample of carers reported high levels of psychological symptoms in terms of depression, anxiety and complicated pre and post death grief. It was evident that the care home transition was itself an extremely upsetting process that may have led to reduced carer tasks and burden but increased mental distress. Although carers were often unaware of available formal grief support, many did not feel they needed this support and were active in seeking support through their social network, consistent with current theories of grief [37, 38]. However, carers would have liked information on available support as knowing where to seek help provided reassurance. The high levels of depression and grief suggest that perhaps the support sought via informal networks was insufficient in meeting emotional needs. Friends and family may not recognise grief during caring [39] or are perhaps unable to reduce levels of distress. This may hinder the grieving process of adapting to and accepting loss [40]. Two months into bereavement carers continued to report high levels of psychological burden, but at seven months this had reduced.

### Physical health

Physical health was consistent with USA based population norms [24], except for those nine carers who provided data 7 months into bereavement who reported poorer physical health. A UK study also found an increased proportion of people frequently visiting their primary healthcare practitioner in the year after the death of a co-residing person with dementia [41].

### Care at the end of life

During interviews, the poor experiences of care reported were alarming. A number of carers described the need to pay considerably higher financial amounts for better quality services, raising concerns about the quality of services available to more deprived members of society. Any need to change service provider is disruptive and impacts on the continuity of care for the person with dementia and creates additional, unnecessary stress for carers.

However, carer satisfaction with EOL on the SWC-EOLD questionnaire indicated moderate satisfaction similar to that reported in studies from the USA and Netherlands [42]. Satisfaction remained stable, however 30% of carers did not feel “fully involved in all decision making” and “would probably have made different decisions if [they] had had more information.” The importance of these items was illustrated by the overarching theme from the qualitative data reflecting the carer’s capacity to understand and influence the experience of EOL care. While information provision about the progression of dementia tended to be poor, carers’ engagement in advance care planning and decision making appeared to improve during hospitalisations and after care home placement. While many described poor quality care in various settings, it was important for carer wellbeing to be able to effect a change in service provider to lead to improved care quality. Two carers reported high levels of guilt and grief into bereavement and this appeared linked to their inability to effect change in care provision combined with a poor knowledge of dementia which made it difficult for them to question staff. There was evidence that changes in their relative’s health status, such as a loss of weight and reduced food intake, were perceived as indicators of poor quality care when they may have reflected the natural progression of dementia. This again highlights the importance of carers understanding common symptoms of EOL in dementia.

### Use of aggressive treatments at EOL

The European Association for Palliative Care’s White Paper recommends avoiding overly aggressive treatments and that the use of antibiotics for life-prolonging effects especially in pneumonia needs to be considered [6]. Some carers in our study held conflicting views and saw the use of intravenous antibiotics and hospitalisation as beneficial and enabling their relative to have additional years of life. This raises an important ethical issue about the extent to which interventions are in the best interests of the family or the person with dementia, particularly when the person with dementia has not had an opportunity to express their preferences. This reinforces the importance of trying to elicit the preferences of the person with dementia at a time when they are able to make informed decisions about EOL care and the potential benefits of discussions about EOL care and advance care planning during the earlier stages of dementia.

### Strengths and limitations

This study used a mixed methods approach with equal importance attributed to qualitative and quantitative findings. Both data types were collected from one longitudinal study, analysed independently and then combined at the point of interpretation and write up to minimise confirmation bias. Quantitative data were limited by the small sample size, particularly into bereavement. However, given the small body of research on carers’ EOL experiences, this study provides an important contribution to the literature. Although the response rate of 52% was acceptable, the sample was selected from a pool of carers who had previously agreed for their relative with dementia to be involved in a larger cohort study [19] and may have been more likely to support research participation. We recruited from a range of care homes in urban and suburban areas across Greater London UK, however we cannot be sure that our sample is typical of family carers of people with advanced dementia. Carers across settings provided assistance with personal care tasks every third day, for an average of 1.4 h per day (our data are biased towards those in a care home). This level of involvement was higher than that of Kiely et al.’s USA study [43] and may explain our high levels of grief. The level of involvement of the carer may be an important factor in determining psychological wellbeing.

Our findings may be influenced by the researcher who spent time each month with the carer. In interviews, some carers stated that they valued interaction with the researcher and that being involved in the study had educated them about EOL care, and helped them to think about their experiences and emotions.

We anticipated a smaller number of participants would be caring for people with dementia at home given that people with advanced dementia tend to reside in care homes and only 5% of people with dementia in the UK die at home [3]. In our sample 17% were caring for the person with dementia at home. However, the small sample size prevents any comparison in outcomes between carers of those living at home compared with those living in a care home. The small sample also prevents any comparison between spouse and child carers which impacts on the grief experience [13].

### Implications

Our findings support the provision of greater information and emotional support for carers at the later stages of dementia. We suggest that carers receive support according to the UK National Institute for Health and Care Excellence (NICE) EOL Quality Statement 14 (Bereavement Support) which recommends that those closely affected by death are offered information about the experience of bereavement and how to access support and that this support may be required before death [44]. Information about dementia progression is needed throughout the course of dementia and provided in a supportive and respectful way focusing on establishing trusting relationships [39]. We also need to understand how we can better support carers through a long and unpredictable disease trajectory which makes it difficult to plan and prepare for end of life. Information booklets can play an important role; almost 50% of current carers reported that they would have wanted to have received a booklet at the time of or soon after diagnosis [45].

Further research could examine whether there are modifiable factors that assist carers to cope with grief. For example, feeling unprepared for death is associated with complicated grief after death [15], and information provision about dementia progression and EOL may be closely tied with carers’ experiences of grief and guilt. Studies that examine how we can better support carers to feel empowered and involved in formal health and social care delivery throughout the progression of dementia could also make a valuable contribution to carer wellbeing.

## Conclusions

Carers report high levels of psychological distress during advanced dementia and in the immediate months into bereavement. However, the experience of EOL care in dementia may be amenable to change with the provision of sensitive and timely information about the natural progression of dementia to family carers. Also, providing regular updates about changes in the health status of the person with dementia and discussing EOL preferences can help families understand the progression of disease and prepare for end of life. The extent to which our findings reflect practice across the UK or internationally warrants further investigation.

## Abbreviations

Brief COPE: Brief Coping Orientation to Problems Experienced
EMI: Elderly Mentally Impaired
EOL: End of life
HADS: Hospital Anxiety and Depression Scale
ICG: Inventory of Complicated Grief
IQR: Interquartile Range
SD: Standard Deviation
SF-12: Short Form 12
SWC-EOLD: Satisfaction with Care at EOL in Dementia Scale
ZBI: Zarit Burden Interview
